# Evaluation of microbial occurrence in reusable robotic instruments for minimally invasive surgery: A pilot study

**DOI:** 10.1371/journal.pone.0300355

**Published:** 2024-04-04

**Authors:** Roy J. Pelzer, Wil C. van der Zwet, Mike M. E. G. Eggen, Ashley Beard, Paul H. M. Savelkoul, Jeanne A. M. C. Dirks

**Affiliations:** Department of Medical Microbiology, Maastricht University Medical Center, and Care and Public Health Research Institute (CAPHRI), Maastricht University, Maastricht, The Netherlands; Universidad Tecnica de Manabi, ECUADOR

## Abstract

In recent decades, minimally invasive surgery has become the favoured surgical technique, with increasing utilisation of robotic surgery to enhance patient outcomes. However, the design complexity of surgical robotic instruments can pose challenges in maintaining adequate cleaning, disinfection and sterilisation—particularly of the device’s interior. In our hospital, robotic instruments are reused for a maximum of ten successive patients, following the manufacturer’s guidelines. To the best of our knowledge, neither the manufacturer nor ISO standards have specified any methods to determine the sterility of robotic instruments after cleaning, disinfection and sterilisation procedures. In a small pilot study, we used a locally developed protocol to evaluate the sterility of 20 da Vinci SI robotic instruments, with the aim of determining whether the recommended cleaning, disinfection and sterilisation process is adequate to achieve safe usage in subsequent patients. None of the 20 instruments showed viable micro-organisms, therefore the robotic instruments were considered sterile, and suitable for re-use. We recommend our protocol to other hospitals, to be used as an essential control element in the assessment of their unique reprocessing technique for robotic instruments.

## Introduction

Robotic surgery has been employed in operating theatres for over 30 years to carry out minimally invasive surgery (MIS) [1, 2]. The da Vinci Surgical System (Intuitive Surgical Inc., Sunnydale, CA, USA) [3] is the most utilised system, having been employed in more than 10 million robotic-assisted surgical procedures as of 2021. The system can be used in a wide range of surgical procedures, including thoracic, breast, colon and rectal, head and neck, gynaecological, urological and general surgery [4]. MIS offers several advantages such as less pain, faster return to oral intake, shorter hospital stay, and improved cosmetic results [5].

**Fig 1:**
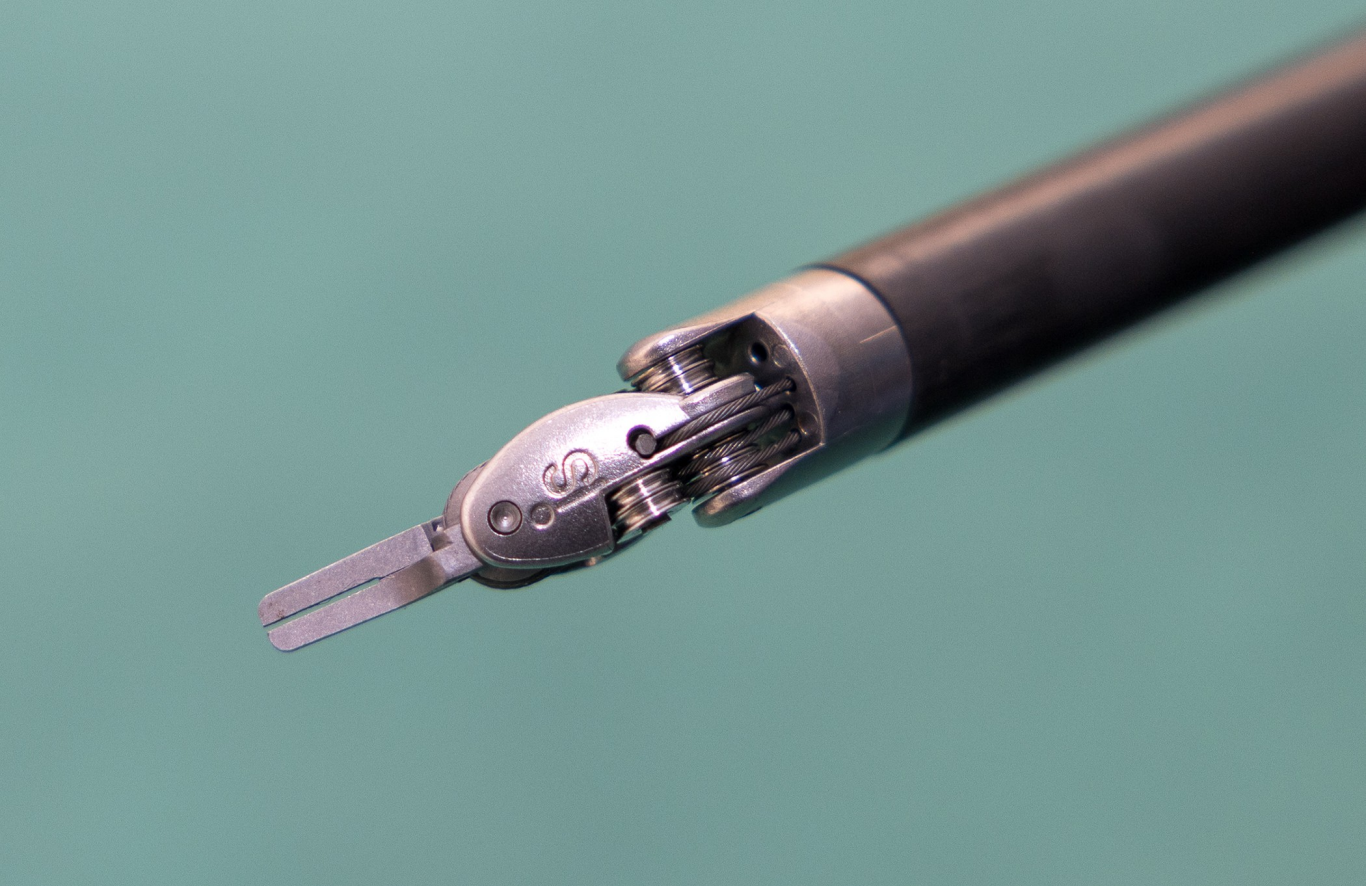
Example of the distal end of a robotic instrument (Cadiere forceps).

The da Vinci Surgical System consists of a console to operate the instrument, the robot itself with several arms and removable surgical instruments [6]. However, these removable surgical instruments are notoriously difficult to clean, especially the interior [7–9]. The removable surgical instruments generally consist of a piece that connects to the robotic arm, a long insulated shaft housing cables that operate the articulated wrist and a distal working end (grasper, scissors, cautery tip, etc. See Fig 1 for an example).

According to the manufacturer, the da Vinci SI instruments can be used up to 10 times, and must be decontaminated after each use [10]. It is imperative that all bioburden is removed to prevent corrosion and instrument damage, as well as the spread of bacteria between patients or staff [11]. However, these robotic instruments cannot be disassembled, which complicates the cleaning process [6]. The thin wires that open and close the working end are particularly problematic, as they draw contaminated material into the shaft [9, 12]. International standards, such as ISO, provide general advice on how to clean and disinfect these instruments, and subsequently test them for sterility. The intricate design of these instruments led us to question whether the process described by the manufacturer and ISO standards is really sufficient to adequately remove microbial residues, particularly from the inside of the instrument. To the best of our knowledge, there is no literature on how to validate the microbiological cleaning process of these instruments, while publications on how to validate cleaning processes that remove protein residues, using either ‘destructive’ or ‘non-destructive’ methods, do exist [6, 13]. Some of these methods testing for protein residues employ ATP measurements, which are also elevated if microbes are present, however there is little correlation between the microbial load and measured ATP levels [14]. The aim of this study was to investigate whether the cleaning, disinfection and sterilisation (CDS) procedure used in our hospital is sufficient to remove all viable microbial residues from such a complex surgical instruments, using both ‘destructive’ and ‘non-destructive’ methods.

## Materials and methods

This study was performed at the Maastricht University Medical Center in the Netherlands, between January 2021 –December 2021. Approximately 250 surgical procedures utilising robotic instruments are carried out annually at our hospital and on average, five instruments are employed per procedure. To validate the CDS procedure, 20 robotic instruments were tested after completion of the standard CDS procedure, and the results were compared to 4 robotic instruments tested <1 hour after patient use.

According to the manufacturer’s instructions, each robotic instrument can be safely used in 10 subsequent patients [10]. In our study, we used robotic instruments that had been used 9 times during MIS. Instead of using the instrument for MIS in a 10^th^ patient, we tested the instrument for microbiological safety.

We studied several da Vinci SI robotic type SH1939 instruments (Intuitive, Sunnyvale California USA): Cadiere forceps, Double fenestrated grasper, Large needle driver, Maryland bipolar forceps, Monopolar cautery spatula, Monopolar curved scissors, ProGrasp forceps and Small clip applier.

### CDS procedure at the Maastricht University Medical Center

After surgery, any tissue or coagulation residues are removed from the tip of the instrument. Additionally, in order to prevent coagulation of blood and tissue, each flush port of the robotic instruments is flushed with at least 20cc of sterile water port immediately after the surgical procedure.

The instruments are subsequently transported to the central sterilisation department within 30 minutes, where any visible contamination is removed from the jaw and jaw components by means of manual pre-cleaning. This cleaning process consists of the application of Polacid cleaning solution (Alpheios, Heerlen, the Netherlands) with a soft nylon brush, followed by flushing of the instrument’s two flush ports with water.

As an additional procedure, not recommended by the manufacturer, the instrument is connected to the Medisafe SI PCF workstation for ultrasonic cleaning of tubular structures (Medisafe, Hertfordshire, UK) using 3E-Zyme (Steris, Leicester, UK), a pH-neutral cleaner. This step is carried out at an average temperature of 43 ˚C for 20 minutes after which a thermal disinfection is performed at 93 ˚C for 4 minutes.

Next, each instrument is placed in a washer-disinfector appliance (PG8528; Miele professional, Gütersloh, Germany) for a 5 minute pre-wash at 46.8°C, followed by cleaning with reverse osmosis water, specifically Neodisher Mediclean Forte (Dr. Weigert, Assen, the Netherlands), for 5 minutes at a temperature of 56 ˚C. Finally, the instrument is thermally disinfected for 5 minutes at 93°C.

As a final step in the CSD process, the instruments are packaged in Steriking Wipak 150 mm, 6" steam packaging (Wipak, Helsinki, Finland) and dry sealed, followed by sterilisation in the Miele steriliser type PS 5662 (Miele Professional, Gütersloh, Germany) at 134˚C for 5 minutes and 18 seconds.

### Microbiological validation

Sampling of the robotic instruments took place in a Laminar Airflow (LAF) cabinet, disinfected with 70% alcohol by a technician dressed in a sterile gown, gloves, and a surgical mask to prevent contamination.

The instruments were sampled by injecting 10 mL of sterile water at a 45 degree angle into the flush port and collecting the outflowing fluid in a sterile jar for culture. If not enough liquid was obtained from the tip, more liquid was obtained from the opening of the flush port by tilting the instrument. This procedure was repeated until at least 5 ml of specimen was collected.

Following collection, 1 mL of each specimen was pipetted into a solution containing 5 mL of Brain Heart Infusion (BHI) and 8 mL of Thioglycollate Medium (Becton Dickinson, Franklin Lakes New Jersey, USA). The tip of the instrument was also removed and cultured in 5 mL BHI broth.

All inoculated media were incubated in an O_2_-incubator at 37°C for 14 days, and assessed daily for visible turbidity. This protocol is similar to the protocol used in our laboratory to culture orthopaedic and vascular implants, to ensure that both fast growing bacteria (eg. *S*.*aureus*, *E*.*coli)* and relatively slow growing bacteria (like *Cutibacterium acnes*) are detected. After the maximum incubation period, or at an earlier time point when visible turbidity occurred, 10 μL of BHI broth was streaked onto a Colombia blood agar plate with 5% sheep’s blood (Becton Dickinson, Franklin Lakes New Jersey, USA) and a chocolate agar plate (Oxoid, Wesel Germany). Likewise, 10 μL of Fluid Thioglycollate Medium was streaked onto a Schaedler agar plate containing Vitamin K1 and 5% sheep’s blood (Becton Dickinson, Franklin Lakes New Jersey, USA). The blood and chocolate agar plates were incubated for 48 hours in an O_2_-incubator set at 37°C. The Schaedler agar plates were incubated under anaerobic conditions at the same temperature and for the same duration. Positive cultures were further characterised using Maldi-Tof MS (Biomerieux, Marcy-l`Etoile, France).

Due to the small number of samples in our pilot study, only descriptive statistical analyses were performed. There were no human participants or samples in this study, and therefore this study did not have the need for medical ethics approval.

## Results

Twenty-four robotic instruments were selected at random for our study. 20 of these were assessed after the CDS procedure and 4 were evaluated before the CDS procedure (immediately after patient use).

Out of the 20 arms that underwent CDS, 19 (95%) were found to be properly sterilised with no growth observed (Table 1). Only one colony of *Staphylococcus capitis* was discovered on the blood agar plate used to culture the BHI broth of the tip and lumen of a Cadiere Forceps instrument after a 14-day incubation period. All the other cultures of this arm were negative (Table 1). The presence of a single colony of *S*. *capitis* in only one medium and on a single agar plate was interpreted as contamination. Therefore, after the CDS procedure, 100% of the robotic wrists were truly culture-negative.

**Table 1:** Culture results per type of instrument.

Type of instrument	N	Growth (with identification)
Before CDS		
Double fenestrated grasper	2	No growth
Maryland Bipolar Forceps	2	Growth in BHI: several types of coagulase-negative *Staphylococcus spp*.
After CDS		
Cadiere Forceps	1	*S*. *capitis*^a^
Large Needle Driver	1	No growth
Maryland Bipolar Forceps	5	No growth
Monopolar Cautery Spatula	4	No growth
Monopolar Curved Scissors	3	No growth
ProGrasp Forceps	1	No growth
Small Clip Applier	5	No growth

^a^ 1 colony on 1 agar plate, most likely contamination.

The four robotic arms tested before CDS were used in four separate patient procedures. Of these, 2 (50%) had positive cultures (Table 1). No pathogenic bacteria were detected, instead the bacteria found were skin bacteria (several types of coagulase-negative Staphylococci). Regrettably, it is not known which procedure the arms were used for and therefore which specific bacteria should have been anticipated.

## Discussion

In this pilot study, we investigated the microbiological safety of 20 Da Vinci SI instruments, which had each been used for 9 patient procedures. Since no standard procedure exists for the microbiological validation of CDS protocols used on these instruments, we followed a local sampling and culture protocol and found no bacterial growth. This was unexpected because of the complex design of the instrument, which includes an open connection between the pliers and the inside of the robotic arm, therefore making cleaning almost impossible. In addition the housing is made of plastic, which conducts heat far less efficiently than metal, which is what most surgical instruments are made of. Despite these factors, we demonstrated that the CDS method used in our hospital, which is in accordance with both ISO standards, is sufficient for safe reuse of these implements in up to 10 patients, as stated by the manufacturer. Further research is needed to establish the safety of re-use after 10 operations, which would increase the sustainability of surgery and decrease medical costs.

As far as we are aware, there are no previous studies investigating the microbiological residues of robotic instruments using culture, while there are several that take protein residues or ATP-measurements into account [13, 15]. Our results are in line with the results of Lopes *et al*. who actively contaminated orthopaedic surgical instruments with bacteria and tested manual or manual followed by automated cleaning and sterilisation [16]. They could not detect viable bacteria after sterilisation, but they could detect soil after automated cleaning and soil/biofilm after manual cleaning. This is in line with our own experience where residual soil can be seen inside the instrument after destruction. Fortunately, this debris appears sterile in our results.

Recently, Heibeyn *et al*. [17] validated a decontamination process of robot arms that they used in the decontamination area of their Central Sterile Supply Department. Although the design differs from robotic arms used for surgery, the risk of residual protein after cleaning is comparable. They optimized the gripper design such that cleaning in a device-specific optimized position achieved results comparable to cleaning the disassembled individual components. There was no microbiological validation of their design. Due to the increasing use of MIS instruments, there is a need for internationally accepted CDS procedures that overcome the challenges of sterilising such instruments and ensure their microbiological safety for re-use. [7–9].

As mentioned before, protein residues and ATP measurements are more commonly used in this type of study. For example, the DaVinci working group has previously described both destructive and non-destructive testing methods, employing the detection of protein residues, that determine the efficacy of cleaning protocols applied to MIS robotic instruments [6]. Chen, *et al*. also examined protein residues when comparing three ‘non-destructive’ methods of determining the cleanliness of da Vinci surgical robotic instruments. From these studies, specific acceptance criteria have even been formulated for protein residues [6, 13]. These studies have also highlighted the barriers to adequately removing such residues from the instrument [8, 9]. ATP measurements are fast and easy to obtain, but may lead to positive results for microbial contamination [14]. ATP measurements on endoscopes have shown a correlation with culture outcomes, but no such data exists yet for robotic instruments [18]. Future research on robotic instruments should incorporate both ATP measurements and culture results, as ATP measurements are faster and easier to obtain [15].

In addition to the manufacturer’s instructions for use, in our hospital the ultrasonic cleaner (Medisafe) is used in the CDS process for extra safety. More and more manufacturer’s now recommend ultrasonic irrigation and cleaning for their instruments [11]. Further research is required to assess whether this step is essential for the CDS procedure since reprocessing without ultrasonic cleaning may yield different results. Moreover, for this study, the robotic instruments were dismantled to obtain accurate cultures from both the interior and exterior of the device. Our findings indicate that there are no apparent benefits to be gained from this methodology, and we suggest that future research explores the non-invasive techniques outlined in this study.

Strict guidelines exist for the sterilisation of medical equipment, with regular validation procedures carried out in accordance with international standards such as ISO 15883, ISO 11737–2:2019 / 17665–1:2006. The aim of this pilot study was to start the development of a standardised microbiological validation process for the CDS procedure on robotic instruments, similar to those already established for protein content [6, 8, 13].

In summary, we present a non-destructive protocol for detecting microorganisms on da Vinci SI robotic instruments. Employing this protocol, we were unable to identify viable microorganisms after following the manufacturer’s instructions for CDS and performing additional ultrasonic cleaning. Our study method is, nonetheless, arduous, and further research should investigate the added significance of ATP measurements in this respect.
